# Development of a Gas-Tight Syringe Headspace GC-FID Method for the Detection of Ethanol, and a Description of the Legal and Practical Framework for Its Analysis, in Samples of English and Welsh Motorists’ Blood and Urine

**DOI:** 10.3390/molecules27154771

**Published:** 2022-07-26

**Authors:** Luke Taylor, Lili Saskőy, Tara Brodie, Vytautas Remeškevičius, Hannah Jayne Moir, James Barker, John Fletcher, Baljit Kaur Thatti, Gavin Trotter, Brian Rooney

**Affiliations:** Faculty of Science, Engineering and Computing, School of Life Sciences, Pharmacy and Chemistry, Kingston University, Kingston upon Thames, London KT1 2EE, UK; l.taylor@kingston.ac.uk (L.T.); k1700497@kingston.ac.uk (L.S.); t.brodie@kingston.ac.uk (T.B.); k1501503@kingston.ac.uk (V.R.); h.moir@kingston.ac.uk (H.J.M.); j.barker@kingston.ac.uk (J.B.); j.fletcher@kingston.ac.uk (J.F.); b.thatti@kingston.ac.uk (B.K.T.); g.trotter@kingston.ac.uk (G.T.)

**Keywords:** HS-GC-FID, ethanol, method validation, gas-tight syringe, method validation

## Abstract

Ethanol is the most commonly used recreational drug worldwide. This study describes the development and validation of a headspace gas chromatography flame ionisation detection (HS-GC-FID) method using dual columns and detectors for simultaneous separation and quantitation. The use of a dual-column, dual-detector HS-GC-FID to quantitate ethanol is a common analytical technique in forensic toxicology; however, most analytical systems utilise pressure-balance injection rather than a simplified gas-tight syringe, as per this technique. This study is the first to develop and validate a technique that meets the specifications of the United Kingdom’s requirements for road traffic toxicology testing using a Shimadzu GC-2014 gas-tight syringe. The calibration ranged from 10 to 400 mg/100 mL, with a target minimum linearity of r2 > 0.999, using tertiary butanol as the internal standard marker. The method has an expanded uncertainty at 99.73% confidence of 3.64% at 80 mg/100 mL, which is the blood alcohol limit for drink driving in England and Wales. In addition, at 200 mg%—the limit at which a custodial sentence may be imposed on the defendant—the expanded uncertainty was 1.95%. For both the 80 mg% and 200 mg% concentrations, no bias was present in the analytical method. This method displays sufficient separation for other alcohols, such as methanol, isopropanol, acetaldehyde, and acetone. The validation of this technique complies with the recommended laboratory guidelines set out by United Kingdom and Ireland Association of Forensic Toxicologists (UKIAFT), the recently issued Laboratory 51 guidelines by the United Kingdom Accreditation Service (UKAS), and the criteria set out by the California Code of Regulations (CCR), 17 CCR § 1220.1.

## 1. Introduction

Ethanol is the most encountered recreational drug in forensic toxicology [1,2]. Due to its depressant effects on the central nervous system (CNS), ethanol causes significant impairment of driving performance [3]. Most countries have set alcohol concentration limits for drivers. In England and Wales, the drink driving limit in blood is set at 80 mg/100 mL (0.08%), with equivalent urine and breath limits of 107 mg/100 mL and 35 μg/100 mL, respectively. These limits are among the highest in the world. By contrast, Scotland and many European countries have set a 50 mg/100 mL ethanol limit in blood [4]. Northern Ireland has also proposed an amendment to the law to lower the prosecution limit to 50 mg/100 mL in blood, along with an additional limit of 20 mg/100 mL for newly qualified drivers and taxi drivers, which is yet to be accepted at the time of publication [5].

In England and Wales, motorists found above the prescribed limit are charged under Section 5 of the Road Traffic Act 1988 [6]. This includes driving, attempting to drive, or being in charge of a motor vehicle on a road or other public place after consuming so much alcohol that the proportion of it in the breath, blood, or urine exceeds the prescribed limit. While most drink driving cases are prosecuted on the basis of evidential breath readings, there is still a requirement for blood or urine analysis in cases in which a breath sample cannot be obtained [7]. To aid law enforcement, rapid, cost-effective, and accurate methods to quantify ethanol in blood and urine are needed. Headspace gas chromatography with flame ionisation detection (GC-FID) is the industry standard alcohol analysis technique, due to its effectiveness at detecting volatile compounds with minimal sample preparation [8,9].

Headspace gas chromatography operates on the principle of Henry’s law—that the concentration ratio of a volatile substance in the gas phase of a closed vessel and in the liquid phase is fixed at a given pressure and temperature. There are a number of different techniques for transferring a sample from the headspace to the chromatography columns [10,11]. These include filled-loop, pressure-balance [12], and direct injection systems [13,14].

The use of headspace gas chromatography for the analysis of ethanol in bodily fluids is not new. Many such methods have been published in the past [15,16]. However, there are differences in approach and regulations between countries, as well as differences in the equipment used. The US state of California regulates road traffic alcohol analysis through its Code of Regulations (CCR), 17 CCR § 1220.1 [17], while in the UK there are no specific regulations relating to this analysis.

PerkinElmer’s pressure-balance headspace sampling system is widely used in the UK for testing road traffic ethanol samples. We “inherited” a Shimadzu gas-tight syringe sampling system—a Shimadzu GC-2014 gas chromatograph equipped with an HTA 200 H headspace autosampler. The main interest of this research was to see how this sampling technique would perform in comparison to the pressure-balance system. Since we did not have access to the pressure-balance system, a direct comparison could not be made, so we assessed the gas-tight syringe system against the California codes and the de facto quality criteria used in England and Wales. The California codes were chosen arbitrarily, as California is a sizeable jurisdiction outside of the UK whose codes we had access to.

## 2. Results

### 2.1. Separation Conditions

**Figure 1:**
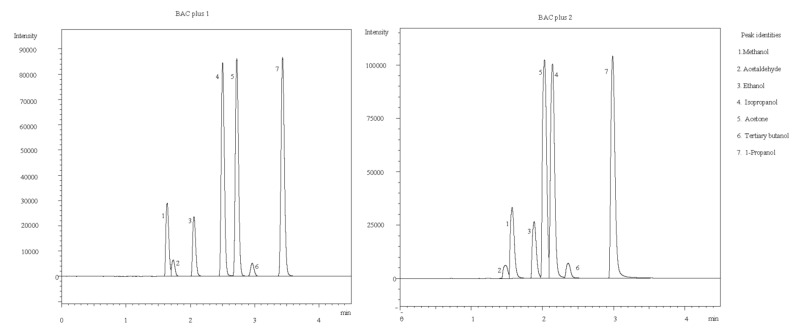
**Separation of ethanol from commonly detected alcohols:** Both columns demonstrated sufficient separation of ethanol from a range of alcohols, including methanol, isopropanol, tertiary butanol, and 1-propanol, in addition to ethanol’s major metabolite acetaldehyde. Separation of ethanol and acetone was achieved on BAC 1, while BAC 2 demonstrated co-elution with acetone at concentrations greater than 10 mg/mL.

The California codes, 1220.1 (a) (2), require that the method be capable of the analysis of ethanol with a specificity that is adequate and appropriate for traffic law enforcement. Figure 1 details the specificity of both columns, and demonstrates that the BAC plus 2 column showed complete separation of ethanol from any of the common interferences that might be encountered in road traffic cases. BAC plus 1 did not entirely separate ethanol from acetone; however, concentrations of acetone of 10 mg/100 mL and below did not interfere with ethanol resolution and/or quantification. It is our understanding that in California the results of one column are used for quantitation, with the results of the other column being used to confirm the identification of ethanol. In England and Wales, the results of both columns are used for quantitation. The precision of the four sub-results (duplicate analysis on dual columns) is checked. Typically, limits of either a %CV of 2.5, or an absolute limit of within +/−2 mg/100 mL (0.002%), are applied. In cases where this limit is not met, due to the presence of interference from acetone, the analysis is repeated, and quantitation is undertaken using the four sub-results from the BAC plus 2 column.

### 2.2. Linearity

Linearity was assessed using calibrators at 10, 20, 50, 100, 200, and 400 mg/100 mL (Table 1). In England and Wales, a minimum permitted R^2^ value of 0.998 is typically applied in road traffic alcohol cases. During validation, 11 separate extractions were analysed; in all analyses the measured R^2^ value in both the BAC 1 and 2 columns was greater than 0.999. In addition, the average residual sum of squares was found to be 0.00336 and 0.00339 for BAC 1 and BAC 2, respectively.

### 2.3. Precision

The intra-run and inter-run precision was assessed using ANOVA. No significant differences were found for the quality controls at 80 and 200 mg/100 mL, so the intermediate precision was calculated as the standard deviation of the set of duplicate results from the 11 batches. Significant differences were found at the value of 20 mg/100 mL, so the intermediate precision was calculated as the square root of the sum of squares of the intra- and inter-run precision. The California codes stipulate a precision limit of +/−5% of the value for alcohol concentrations above 0.100% (100 mg/100 mL) and +/−0.005% (5 mg/100 mL) for alcohol concentrations lower than 0.100%. The precision of our method was found to be +/−0.0006% at 200 mg/dL, +/−0.0012% at 80 mg/100 mL, and +/−0.0031% at 20 mg/100 mL (Table 2).

### 2.4. Accuracy

The California codes stipulate an accuracy limit of +/−5% of the value for alcohol concentrations above 0.100% (100 mg/100 mL) and +/−0.005% (5 mg/100 mL) for alcohol concentrations lower than 0.100%. The accuracy of our method was found to be +/−0.00116% at 200 mg/100 mL, +/−0.00048% at 80 mg/100 mL, and +/−0.00089% of the value at 20 mg/100 mL (Table 2). The bias was evaluated using a *t*-test incorporating the uncertainty of the target—the certified reference material uncertainty—into the measurement uncertainty. The bias was found not to be significant for the quality control samples at 80 mg/100 mL and 200 mg/100 mL. There was significant bias for the control samples at 20 mg/100 mL. The method uncertainty was calculated by combining the method intermediate precision with the uncertainty of the bias measurement as the square root of the sum of the squares.

### 2.5. Uncertainty

The method has a standard uncertainty at 200 mg/100 mL of 0.65%, at 80 mg/100 mL of 1.2%, and at 20 mg/100 mL of 1.77%, giving expanded uncertainties of 1.95% (or 3.88 mg/100 mL), 3.64% (or 2.89 mg/100 mL), and 5.31% (or 1.11 mg/100 mL), respectively, at a 99.73% confidence. In England and Wales, 6%, or 6 mg/100 mL—whichever is greater—is subtracted from the measured value as an allowance for measurement uncertainty. The result is then reported as being “not less than” this subtracted figure.

**Figure 2:**
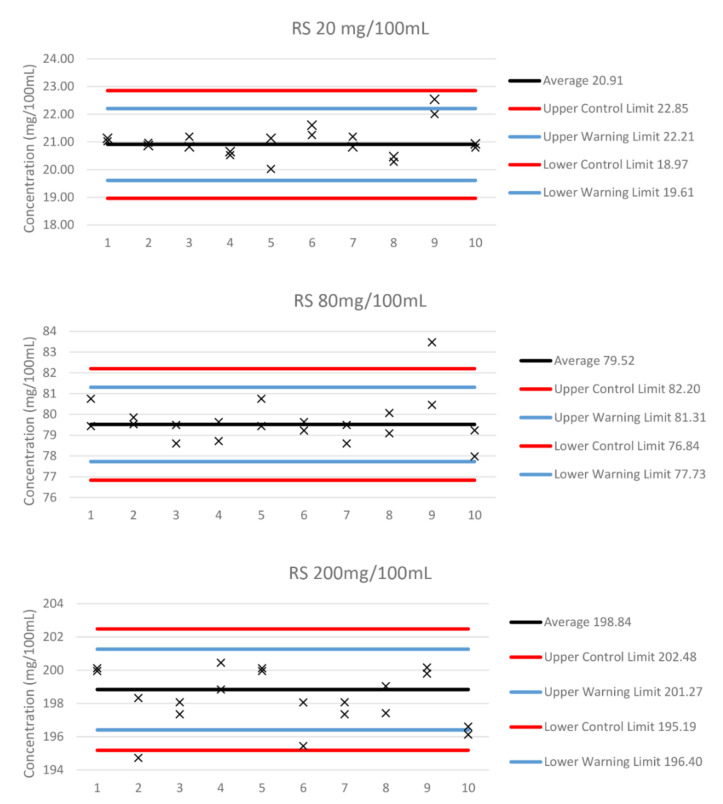
**Determination of method uncertainty using the certified reference material**: The method has a standard uncertainty at 200 mg/100 mL of 0.65%, at 80 mg/100 mL of 1.2%, and at 20 mg/100 mL of 1.77%, giving expanded uncertainties of 1.95% (or 3.88 mg/100 mL), 3.64% (or 2.89 mg/100 mL), and 5.31% (or 1.11 mg/100 mL), respectively, at a 99.73% confidence.

Figure 2 shows the quality control charts of the three different QC levels from 10 batches run on different days in duplicate analysis, once the method validation had been completed. The lower and upper warning and control limits in this method were defined as +/−2 and +/−3 standard deviations of the mean of validation QC values. The California codes recommend the acceptable limits to be set as +/−10 mg/100 mL of the mean value determined from 20 replicate analyses.

### 2.6. Limit of Detection and Quantification

Ethanol was qualitatively detected at concentrations of 5 mg/100 mL with a signal-to-noise ratio greater than 3:1—concentrations approximately half that of the limit of quantitation. Qualitative assessment of the 5 mg/100 mL concentrations from four separate extractions on separate days showed an average ethanol concentration of 6.1 mg/100 mL (detected below the lowest calibrator) and an average ethanol peak area of 2162. The limit of quantitation (LOQ) was set at the lowest calibrator sample—10 mg/100 mL. This methodology has been deployed in range of sample types, including research studies [18], proficiency test trials, and road traffic casework analysis. From the analysis of 30 blood samples analysed over a period of approximately 18 months, the range of blood alcohol concentrations was 6.55 to 230.97 mg/100 mL, the average standard deviation was 1.55, the average %CV value was 2.96, and the median concentration was 43.44 mg/100 mL. Representative chromatograms are detailed in Supplementary Figure S1.

## 3. Discussion

This study details a gas-tight syringe GC-FID method that is capable of quantifying ethanol in blood samples taken in road traffic cases. There is no specific regulation of road traffic alcohol analysis in England and Wales, save for the requirement for methods used by prosecution laboratories to be ISO-17,025-accredited. To varying degrees, laboratories use methods and quality acceptance criteria based on those of the now-closed Forensic Science Service. Our methodology meets the general laboratory criteria as described by the United Kingdom and Ireland Association of Forensic Toxicologists (UKIAFT) [19] and the California Code of Regulations for the quantification of ethanol in road traffic samples.

This analytical technique utilises two separate columns and detectors; the columns chosen were the Restek BAC-1 and BAC-2, and each analytical result is the mean of the four sub-results arising from duplicate analysis on dual columns. The spread of those sub-results was limited to 2.5% at and above 80 mg/100 mL, and 2.5 mg/100 mL below 80. The method uses sodium metabisulphite as an antioxidant, but does not use a salting-out agent. The volume of internal standard added to each 100 µL sample is 1 mL; this floods the sample and prevents salting-out that may occur at lower volumes.

To the best of our knowledge, this publication describes the first dual-column/detector gas-tight syringe HS-GC-FID method that can accurately quantitate ethanol concentrations in blood samples to a forensic standard. Other studies have utilised the GC-FID pressure-balance system [20,21,22]. Our results compare favourably with a 2020 study by Mihretu et al., who detailed a HS-GC-FID technique to detect BAC using a PerkinElmer Clarus 500 pressure-balance injection. This technique had a correlation coefficient of r^2^ = 0.993, and a precision (repeatability) and intermediate precision of 27% and 11%, respectively. This technique also displayed a limit of detection of 9.9 mg/100 mL, and did not use dual-column analysis—a requirement for the forensic testing of road traffic alcohol samples [20].

A correlation coefficient (r^2^) limit of 0.9980 was applied to the linear calibration curves. The limit of quantitation (LOQ)—the lower end of the calibration—was 10 mg/100 mL (0.01%). This method utilises three different concentrations of quality controls: 20 (0.020%), 80 (0.080%), and 200 mg/100 mL (0.200%). The 20 mg/100 mL concentration is used to assess the lower blood alcohol limit for toxicology cases involving aviation staff, and to ensure that quality is maintained below the legal limit should back-calculations (retrograde calculations) be required. The QC at 200 mg/100 mL is used to demonstrate the method’s performance above the 80 mg/100 mL legal limit, as increased sanctions are applied to motorists convicted of driving with BACs significantly above that limit. At present, the magistrates’ court sentencing guidelines [23] specify a fine and a minimum driving ban of 12 months for BACs between 81 and 137 mg/100 mL (0.081 to 0.137%) (the offence is exceeding the limit of 80). A fine and a minimum ban of 17 months are imposed at BACs between 138 and 206 mg/100 mL (0.138 to 0.206%), while a community order and a minimum ban of 23 months are imposed at BACs between 207 and 275 mg/100 mL. A custodial sentence of up to 12 weeks may be imposed in addition to a minimum 29-month ban for BACs between 276 and 345 mg/100 mL (0.276 and 0.345%) and above. The starting sanctions are increased for a range of conditions, including prior convictions and aggravating factors such as carrying passengers.

The QCs are assessed using QC charts with limits based on the method precision, rather than the blanket +/−10 mg/100 mL (0.010) specified in the California codes. Those limits are shown in Figure 2. The California codes require mean QC values to be determined as the mean of at least 20 replicate analyses, at a rate of no more than two analyses per day. In the UK, no such specification exists. We assessed the QCs over 11 batches, with samples run in duplicate, as this is the specification set for Road Traffic Act Section 5 A: drug-driving. The 200 mg/100 mL QC chart shows that the limits are too narrow. Even the sorts of batch numbers specified by the California codes, along with the requirements for Section 5 A, may be insufficient to properly assess the method’s performance. The precision calculated during validation is smaller than it appears to be after validation. We intend to re-assess the QC charts, along with other data, once sufficient data have been collected.

Carryover and matrix interferences were assessed by running blank internal standard samples after the highest calibrator and between duplicate samples and quality controls in every batch. No carryover was observed under the conditions described above. This complies with the requirements for laboratory testing set out by UKIAFT and the California codes.

Results in England and Wales are reported as a subtracted “not less” than value: 6 mg/100 mL, or 6%—whichever is higher—is subtracted from the analytical result. An analytical result of 87.15 mg/100 mL, for example, would be reported as “the sample contains alcohol at a concentration of not less than 81 mg/100 mL”. This subtraction is an allowance for analytical uncertainty. This common approach is used by all laboratories in the UK, and ensures consistency between laboratories. The value of 6 mg/100 mL or 6% is based on the precision of the FSS analytical method introduced in the early 1970s. It far exceeds the uncertainty of modern methods. In the present study, the combined expanded uncertainty (at 99.73%) of our method at 80 mg/100 mL was found to be 2.89 mg/100 mL. In 2010, Sir Peter North recommended the use of 3 mg/100 mL or 3%—whichever is higher—as the subtraction that should be made to allow for analytical uncertainty. However, without regulation, and with the 6 mg or 6% over 100 mg subtraction present in case law, there is no obvious way for this to be implemented.

## 4. Materials and Methods

### 4.1. Chemicals and Reagents

Ethanol standard solutions were purchased from Cerilliant at concentrations of 10, 20, 50, 100, 200, and 400 mg/100 mL (Austin, TX, USA). The certified reference material—aqueous ethanol—was purchased from LGC (Teddington, UK) at concentrations of 20, 80, and 200 mg/100 mL. Anhydrous tertiary butanol and sodium metabisulphite were purchased from Fisher Scientific (Red Rock, CO, USA). Propan-1-ol, propan-2-ol, methanol, acetaldehyde, and acetone were obtained from Fisher Scientific (Red Rock, CO, USA)—all analytical grade. Deionised water was generated on-site using a Sartorius Arium™ advance EDI Water System, ASTM Type 2. Defibrinated horse blood was obtained from TCS Biosciences LTD (Buckingham, UK). Human blood samples were collected on-site from volunteers. The sample collection involving human participants was in accordance with the institutional research committee and the 1964 Helsinki Declaration, or comparable ethical standards. Furthermore, informed consent was obtained from all individuals who donated their samples for testing for either research or casework testing. The research samples collected were approved by the Faculty Research Ethics Committee (FREC) of Kingston University London (ethics code: 1819063.1) Proficiency testing samples were sourced from LGC’s Forensic Blood Toxicology QUARTZ scheme.

### 4.2. Instrumentation

The headspace was sampled using an HTA 200 H headspace autosampler fitted with a gas-tight syringe. The chromatographic analysis was carried out on a Shimadzu GC-2014 gas chromatography system equipped with two FIDs. RTx-BAC 1 (30 m, 0.32 mm ID, 1.8 um) and RTx-BAC 2 (30 m, 0.32 mm ID, 0.6 um) columns (Restek, Bellefonte, PA, USA) were connected to the inlet using a Universal Y tight-press connector. Helium was used as the carrier gas at a column flow rate of 2.78 mL/min. Hydrogen was used as the FID fuel source, compressed air was used as the FID flame oxygen supply, and nitrogen was used as the makeup gas. During method development, investigations into the optimal conditions for efficient and thorough separation of ethanol, tert-butanol, and acetaldehyde were prioritised. The method optimisation involved the modification of GC oven temperature, pressure, and sample conditioning time. Our investigations assessed method performance with a range of pressures from 150 to 65 kPa; in addition, we also researched the effects of modifications in oven temperature between 40 and 50 °C and sample incubation times of 5, 10, 15, and 20 min. Our results indicated that the optimal parameters selected for this method were 40 °C GC oven temperature, a 5-minute conditioning time, and an 85 kPa pressure. The instrument parameters are detailed in Table 3. Shimadzu GC solutions software was used for the data processing.

### 4.3. Sample Preparation

An internal standard (ISTD) solution was prepared by adding 25 μL of tertiary butanol to 500 mL of distilled water, with 2.5 g of sodium metabisulphite (antioxidant). Calibrators, quality controls (QCs), and samples were prepared in duplicate by adding 100 μL of sample to 1 mL of internal standard solution in labelled 20 mL headspace vials. Pipetting was carried out using Gilson Pipetman G 20–200 μL and 2–20 μL adjustable-volume pipettes, a Gilson Microman E 10–100 μL positive displacement pipette, and a VWR 100–1000 μL adjustable-volume pipette.

### 4.4. Headspace GC-FID Method

The prepared samples were conditioned according to the headspace sampling parameters (Table 3). The optimised separation conditions consisted of 40 °C GC oven temperature, a 5-min equilibration time with shaking, and an 85 kPa pressure. The syringe was heated to 70 °C, and a 1 mL sample was injected onto the columns. These parameters allowed for a sub-five-minute sample run time with a full resolution and separation of all compounds.

## 5. Conclusions

The method meets the requirements for our analysis—that is, the analysis of “B” samples. In the UK, a single specimen must be taken. That specimen is split into two, and drivers may request to be provided with one of the parts—the B sample—for independent analysis. Although no direct comparison was made with other headspace sampling techniques, such as loop-filling and pressure-balance systems, this gas-tight syringe method meets the requirements for the alcohol analysis of road traffic blood samples taken in England and Wales, and meets the specifications set in the California Code of Regulations. Gas-tight syringe headspace sampling is a viable alternative sampling technique.

## Figures and Tables

**Table 1:** **Coefficients of determination for dual-column, dual-detector GC-FID analysis of ethanol**: The R^2^ values on both the BAC 1 (column A) and BAC 2 (column B) columns in all validation batches exceeded the minimum requirement of 0.998 for road traffic toxicology testing.

Linearity		
Batch Number	Linearity Column A	Linearity Column B
1	0.99995	0.99995
2	0.99998	0.99998
3	0.99997	0.99995
4	0.99998	0.99995
5	0.99976	0.99974
6	0.99997	0.99998
7	0.99972	0.99975
8	0.99963	0.99983
9	0.99962	0.99997
10	0.99991	0.99993
11	0.99986	0.99986
Average	0.99985	0.99990
Standard deviation	1.42059 × 10^−4^	9.09551 × 10^−5^
CV%	0.01421	0.00910

**Table 2:** Summary of the precision and accuracy of the validation results from 11 batches in mg/100 mL. No significant differences were found at 80 and 200 mg/100 mL—the two limits critical in road traffic toxicology casework.

Precision and Accuracy			
	20 (mg/100 mL)	80 (mg/100 mL)	200 (mg/100 mL)
**Mean**	20.889	79.520	198.836
**Precision**	0.612	1.125	3.104
**Accuracy**	0.892	0.480	1.164

**Table 3:** **Instrumentation Setup**: GC-FID parameters for the dual-column detector system.

Parameter	Value	
**Inlet temperature (°C)**	110	
**Injection mode**	Split	
**Pressure (Kpa)**	85	
**Column flow (mL/min)**	2.78	
**Linear velocity (cm/s)**	42.30	
**Purge flow (mL/min)**	3.00	
**Split ratio**	5.00	
**Oven temperature (°C)**	40, isothermal	
**Detector temperature (°C)**	280	
**Analysis time (min)**	4	
**Headspace oven temperature (°C)**	60	
**Syringe temperature (°C)**	70	
**Fill volume (mL)**	1.75	
**Sample volume (mL)**	1.00	
**Incubation time (min)**	5.00	
**Sample speed (mL/min)**	5.0	
**Shaker time (min)**	0.50 on	0.10 off
**Injection speed (mL/min)**	80	

## Supplementary Materials

The following supporting information can be downloaded at: https://www.mdpi.com/article/10.3390/molecules27154771/s1, Figure S1: Representative chromatograms of (A) Ethanol negative control samples with tertiary butanol internal standard, (B) QC samples spiked with 20 mg/100 mL of ethanol and (C) a casework samples detected at 79 mg/100 mL.

## Sample Availability

Samples are not available from the authors.
