# Supplementary material for: Development of a Gas-Tight Syringe Headspace GC-FID Method for the Detection of Ethanol, and a Description of the Legal and Practical Framework for Its Analysis, in Samples of English and Welsh Motorists’ Blood and Urine
Source: Molecules. 2022 Jul 26;27(15):4771. doi: 10.3390/molecules27154771 (PMC9331811; doi:10.3390/molecules27154771)
Supplement: Supplementary file 1 [file molecules-27-04771-s001.zip › molecules-1799563-supplementary.pdf]

## Supplementary Figure S1

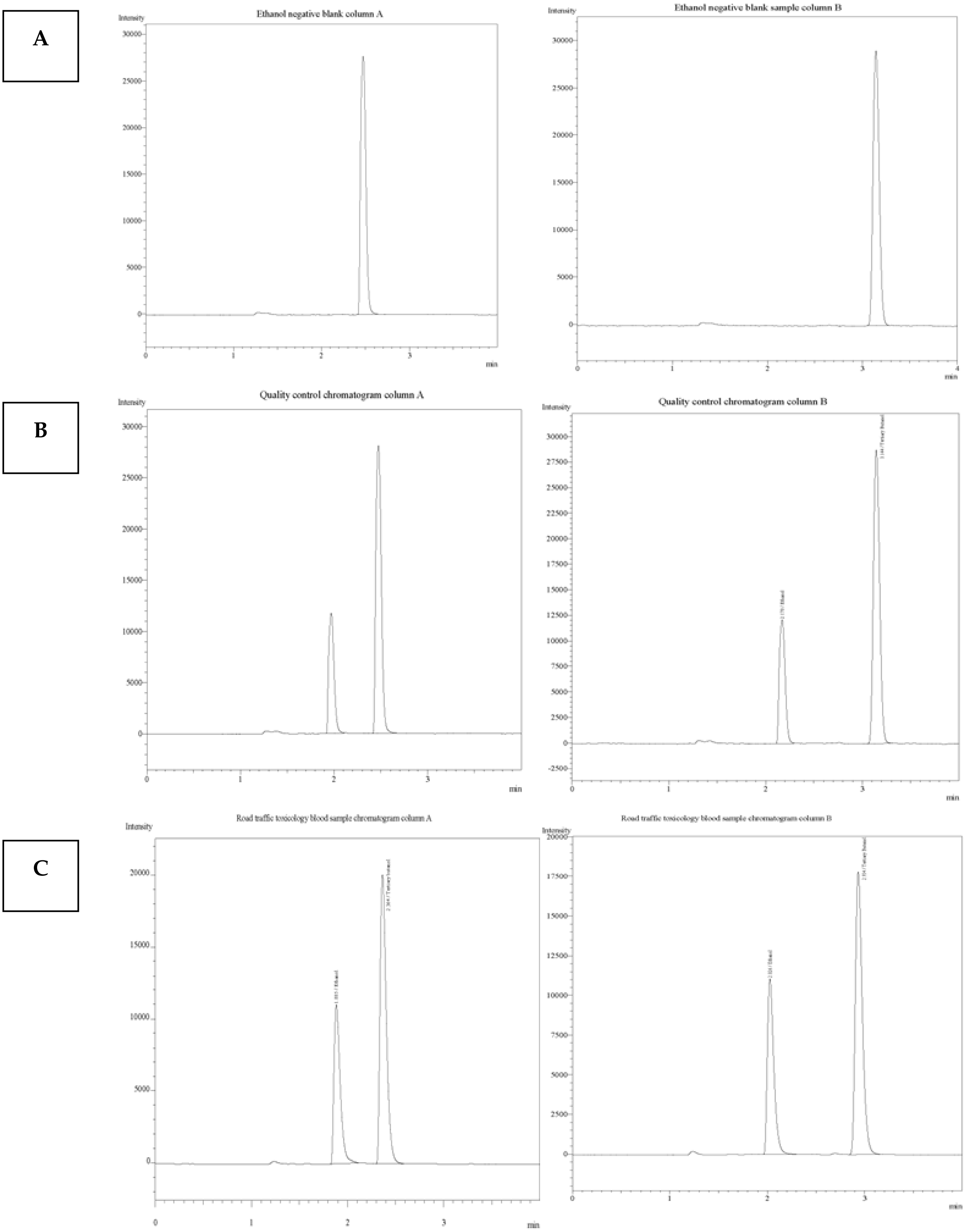


**Supplementary Figure S1**: Representative chromatograms of (A) Ethanol negative control samples with tertiary butanol internal standard, (B) QC samples spiked with 20 mg/100mL of ethanol and (C) a casework samples detected at 79mg/100mL.
